# Effect of 1-DNJ on Oxidative Stress-Induced Apoptosis in Porcine Ovarian GCs Through Modulation of the PERK-ATF4/MFN2 Signaling Pathway

**DOI:** 10.3390/antiox14040456

**Published:** 2025-04-11

**Authors:** Wenwen Xing, Mengxuan Li, Binbin Wang, Lele Huo, Wanru Tian, Fangcai Ge, Manman Shen, Liumei Sun, Jiying Liu, Shali Yu

**Affiliations:** 1 Jiangsu Key Laboratory of Sericultural and Animal Biotechnology, School of Biotechnology, Jiangsu University of Science and Technology, Zhenjiang 212100, China; 221211802115@stu.just.edu.cn (W.X.); 212211801208@stu.just.edu.cn (M.L.); 231211802126@stu.just.edu.cn (B.W.); 241211802126@stu.just.edu.cn (L.H.); 241211802109@stu.just.edu.cn (W.T.); 212211801123@stu.just.edu.cn (F.G.); mms@just.edu.cn (M.S.); sun.liumei@just.edu.cn (L.S.); 2 Key Laboratory of Silkworm and Mulberry Genetic Improvement, Ministry of Agriculture and Rural Affairs, Sericultural Scientific Research Center, Chinese Academy of Agricultural Sciences, Zhenjiang 212100, China; 3 Department of Occupational Medicine and Environmental Toxicology, Nantong Key Laboratory of Environmental Toxicology, School of Public Health, Nantong University, Nantong 226019, China

**Keywords:** ovarian granulosa cells, oxidative stress, 1-DNJ, endoplasmic reticulum stress, mitochondrial dysfunction, mitochondria-associated endoplasmic reticulum membrane

## Abstract

Oxidative stress (OS) is regarded as a major contributor to granulosa cellapoptosis in ovarian disease. 1-Deoxynojirimycin (1-DNJ), a naturally occurring plant alkaloid, exhibits antioxidant, anti-inflammatory, and metabolism-modulating properties. Mitochondria and endoplasmic reticulum (ER), crucial organelles regulating oxidative balance, interact through mitochondria-associated endoplasmic reticulum membranes (MAMs) for signaling and molecular exchange. However, it remains unclear whether 1-DNJ attenuates oxidative damage in ovarian granulosa cells (GCs) via MAMs-mediated ER–mitochondria crosstalk, which needs further exploration. This study aimed to investigate the mechanisms by which 1-DNJ affects oxidative damage and apoptosis induced by OS in porcine follicular GCs by regulating mitochondrial function, MAMs, and ER interactions. Here, we found that GCs suffered from OS, accompanied by the up-regulation of ROS and MDA, alongside reduced activity of antioxidant enzymes (CAT and T-SOD). Further studies revealed that the up-regulation of MAMs proteins (MFN2, MCU, and VDAC1) and pro-apoptosis proteins (BAX and Cleaved-capase3), along with increased mitochondrial ROS and Ca^2+^ levels, led to the down-regulation of MMP and ATP content. These, in turn, triggered mitochondrial dysfunction, and MAMs destabilization, and subsequent apoptosis. Additionally, the up-regulation of the protein levels of P-PERK/PERK, GRP78, ATF4, and CHOP protein expression activated the PERK-ATF4 signaling pathway, which triggered endoplasmic reticulum stress (ERS). Conversely, 1-DNJ alleviated H_2_O_2_-induced mitochondrial and MAMs dysfunction and ERS, which in turn attenuated apoptosis. Further, ATF4 knockdown inhibited MFN2 protein expression, which attenuated H_2_O_2_-induced MMP inhibition, Ca^2+^ overload, ROS production, and mitochondrial damage. In summary, 1-DNJ mitigated OS-induced mitochondrial dysfunction in GCs and regulated ER–mitochondrial communication through MAMs, reducing OS-induced apoptosis. The present study demonstrates that 1-DNJ protects ovarian GCs from OS-induced damage by modulating ER and mitochondrial homeostasis through MAMs, offering new perspectives and a theoretical basis for the treatment of ovarian diseases.

## 1. Introduction

The ovary, as the core of the female reproductive system, plays a decisive role in maintaining female reproductive health and endocrine balance [1]. The ovary is not only responsible for the maturation of germ cells and ovulation to ensure fertility but also maintains the stability of the endocrine system through hormone secretion [2]. However, due to long-term external environmental, stressful, or intracellular factors in the body, the ovary’s ability to scavenge reactive oxygen species (ROS) can be impaired, which disrupts the dynamic balance of the body’s oxidative and antioxidative systems, triggering endocrine disorders, and thus inducing a variety of ovarian diseases, such as polycystic ovary syndrome (PCOS) and premature ovarian failure (POF) [1,3,4]. As one of the most important cell populations in the ovary, granulosa cells (GCs) provide essential factors (e.g., hormones and growth factors) and a microenvironment for oocyte development, maturation, and ovulation [5]. Follicular atresia plays a crucial role as a selective mechanism during follicular development in mammalian ovaries [6], and the apoptosis of GCs is a hallmark of this process. It has been found that oxidative stress (OS) induces apoptosis in ovarian GCs, leading to follicular atresia, and affecting the follicular maturation and ovulation process, ultimately leading to the loss of ovarian function [4]. Therefore, an in-depth understanding of how OS affects the mechanism of GCs apoptosis is important for unraveling the mechanism of ovarian disease development and developing new therapeutic strategies.

Mitochondria, as crucial organelles within GCs, not only produce ATP to provide energy to cells through oxidative phosphorylation but also regulate calcium homeostasis, ROS production and clearance, apoptosis, and necrosis [7]. Excessive ROS accumulation induces mitochondrial OS, resulting in mitochondrial dysfunction. This dysfunction also leads to the release of numerous pro-apoptosis factors from mitochondria, which in turn induces the caspase cascades [8], ultimately impairing cell biology and abnormal follicular development, as well as ovarian disease [4,9]. It was found that, in the induced POI model, ROS expression was increased, mitochondrial membrane potential (MMP) and ATP levels were down-regulated, and mitochondrial morphology was impaired [10]. In addition, OS induced by H_2_O_2_ in chicken ovarian GCs leads to dysfunctions such as the perturbation of calcium homeostasis and depolarization of the MMP, which trigger apoptosis [11]. Given that mitochondrial dysfunction may affect the energy supply and maturation of oocytes, this in turn affects reproductive health. Therefore, targeting OS pathways to mitigate mitochondrial dysfunction is a key and promising therapeutic strategy.

The endoplasmic reticulum (ER) is an important site for protein synthesis, secretion, folding, maturation, and translation in eukaryotic cells. However, various physiological and pathological factors such as nutritional deficiencies, disturbances in Ca^2+^ metabolism, OS, and viral infections can lead to the accumulation of misfolded or unfolded proteins in the lumen, inducing endoplasmic reticulum stress (ERS) [12]. In response to ERS, cells initiate the unfolded protein response (UPR), an adaptive mechanism that primarily relies on ER transmembrane sensors: RNA-dependent protein-like kinase (PERK), inositol-requiring enzyme 1-α (IRE1-α), and activating transcription factor 6 (ATF6) [13]. ERS plays an important role in female reproduction, including follicular development and atresia, early embryonic development and implantation, placenta formation, labor, and fetal growth restriction [13,14]. ERS triggers ovarian atresia and GC apoptosis by mediating the apoptotic pathway, which in turn affects reproductive health [14]. In addition, OS induced by H_2_O_2_ leads to the production of excess ROS, which not only impairs disulfide bond formation and proper protein folding, but also further activates UPR signaling and exacerbates ERS [15]. H_2_O_2_ induces ERS to activate the PERK-ATF4-CHOP pathway, which plays a key role in the transition from ERS to mitochondrial apoptosis. CHOP, a key protein in this pathway, inhibits the expression of Bcl2 family proteins and promotes calcium transfer from the ER to mitochondria. Consequently, ERS triggers mitochondrial apoptosis by activating apoptosis-associated proteins such as BAX and Caspases [16]. In summary, there is a close connection between ERS and OS, which interact with each other and jointly affect cellular redox homeostasis, protein folding, and cell survival or death. Therefore, understanding and regulating ERS is important for maintaining cellular homeostasis and developing treatments for related diseases.

Mitochondria and the ER are functionally interconnected organelles, and the contact sites are mitochondria-associated endoplasmic reticulum membranes (MAMs) [17]. The main functions of MAMs are to promote Ca^2+^ transport, ER membrane remodeling, mitochondrial dynamics regulation, signaling transduction, and apoptosis regulation [17]. The transportation of Ca^2+^, a second messenger, is the most important and fundamental regulatory function of MAMs, e.g., Ca^2+^ can influence hormone secretion and oocyte maturation [18]. Notably, Ca^2+^ transfer from the ER to mitochondria via MAMs is a key step in programmed cell death induction by certain chemicals [19]. Therefore, elucidating the corresponding molecular mechanisms of ERS and mitochondrial dysfunction may provide new therapeutic strategies to prevent follicular atresia.

1-Deoxynojirimycin (1-DNJ), a naturally occurring alkaloid, is found in plants and microorganisms such as mulberry, mistletoe, topiary plants, and Bacillus, but mulberry leaves are currently the main source of natural 1-DNJ [20,21]. This compound has multifaceted bioactivities, particularly in modulating OS and the immune response [22]. Mechanistically, 1-DNJ can improve cardiac function by remodeling the mitochondrial cristae in cardiomyocytes of mitochondrial-type hypertrophic cardiomyopathy (HCM) through optic atrophic protein 1 (OPA1)-dependent oligomerization, thereby restoring mitochondrial morphology and function [23]. In addition, 1-DNJ is involved in the regulation of reproduction in animals. It improves fertility by reducing obesity and obesity-induced inflammation in male animals [24]. Mulberry leaf extract reduces abdominal fat deposition in female broilers, a reduction that indirectly affects their fertility [25]. However, despite its known bioactivity, 1-DNJ has been less extensively studied in pigs, and its potential effects on apoptosis induced by oxidative damage in porcine ovarian GCs remain unexplored. Therefore, this study selected porcine GCs as the research subject and established an H_2_O_2_-induced OS model to investigate the following: (1) whether 1-DNJ can affect OS-induced redox imbalance, ERS response, mitochondrial dysfunction, and apoptosis; and (2) whether these effects occur through an MAMs-mediated regulation of H_2_O_2_-induced ERS, mitochondrial OS, and apoptosis. This study aimed to provide a new direction for the treatment of ovarian OS-related diseases in female animals.

## 2. Materials and Methods

### 2.1. Isolation and Culture of Porcine Ovarian Granulosa Cells

Fresh ovaries were obtained from healthy 6-month-old three-way crossbred sows (body weight 110–120 kg) provided by the Nanjing Zhushun Biotechnology Company (Nanjing, China), and all feeding conditions were normal before slaughter. Seventy ovaries samples were collected, placed in 37 °C saline containing 1% penicillin/streptomycin (Beyotime, Shanghai, China), and transported to the laboratory within 2 h. Follicular fluid was extracted from healthy follicles with a diameter of 3–5 mm using a 22-gauge needle and centrifuged at 800× *g* for 5 min. The objective to remove supernatant liquid and preserve cell precipitation. The precipitated cells were washed twice with DMEM/F12 (Gibco, Courtaboeuf, France) and centrifuged at 800× *g* for 5 min. In the end, the cells were incubated with DMEM/F-12 (1:1) containing 1% penicillin–streptomycin and 10% fetal bovine serum (Thermo Fisher Scientific, Shanghai, China) at 37 °C in a 5% CO_2_ incubator for 48 h. Then the cells were washed three times with phosphate-buffered saline (PBS (BasalMedia, Shanghai, China)), added to DMEM/F12 containing 1% penicillin–streptomycin and 10% fetal bovine serum, and placed in an incubator containing 5% CO_2_ at 37 °C for 24 h.

H_2_O_2_ (30% purity; CAS No. 7722-84-1, Sinopharm, Beijing, China) was added into DMEM/F-12 culture medium to prepare a 1 M storage solution, which was subsequently diluted 1:100 in fresh DMEM/F-12 to obtain a 10 mM solution. Final experimental concentrations were achieved through further dilution with the complete medium.

For the oxidative stress alleviation model, 1-DNJ (≥98% purity; CAS No. 19130-96-2, Anergy Chemistry, Shanghai, China) was dissolved in dimethyl sulfoxide (Sangon Biotech, Shanghai, China) to prepare a 100 mM stock solution. For use, the stock solution was diluted in a ratio of 1:1000 to 100 μM using complete medium. Finally, the diluent was added to the complete medium to prepare the working concentration required for the experiment.

### 2.2. Cellular Viability Assay

GCs were seeded in 96-well plates at a density of 1 × 10^4^ cells/well and cultured in a 5% CO_2_ incubator at 37 °C for 24 h. After the treatment of GCs, the CCK8 reagent (BL1055C, Biosharp, Guangzhou, China) was added to each well. After 2 h incubated at 37 °C, the optical density at 450 nm was measured using a microplate spectrophotometer (BioTek, Winooski, VT, USA). Cellular viabilities were calculated as a percentage of the control.

### 2.3. Analysis of Intracellular ROS

ROS levels in GCs were detected using the fluorescent probe DCFH-DA (CAS No. S0034S, Beyotime, Shanghai, China). Briefly, after the treatment of GCs, 500 µL/well of 10 µM DCFH-DA was added to 12-well plates, and cells were incubated for 20 min at 37 °C, then washed three times with serum-free medium, and observed using an inverted fluorescence microscope (Olympus, Tokyo, Japan).

### 2.4. qRT-PCR

After the treatment of GCs, total RNA was extracted using RNAiso Plus (TaKaRa, Dalian, China), and cDNA was synthesized using a PrimeScript™ RT Kit with gDNA Eraser (CAS No. RR047A, TaKaRa, Dalian, China), according to the kit’s instructions. For quantitative PCR, each 10 µL reaction contained 1 µL of template cDNA (200 ng/µL), 0.3 µL each of the upstream and downstream primers (10 µmol/L), 5 µL of ChamQ SYBR qPCR Master Mix (CAS No. Q311-02, Vazyme, Nanjing, China), and 3.4 µL of DEPC-treated water. The reaction conditions were as follows: 40 cycles of 95 °C for 30 s, 95 °C for 10 s, and 60 °C for 30 s (Bio-Rad, Hercules, CA, USA). Relative gene expression was analyzed by the 2^−ΔΔCt^ method using GAPDH as an internal reference gene. The primer information is shown in Table 1.

### 2.5. Western Blotting (WB)

Total proteins were collected using PMSF (CAS No. ST2573, Vazyme, Nanjing, China) and RIPA lysis buffer (CAS No. P0013B, Beyotime, Shanghai, China) at a ratio of 1:100. The protein concentration was determined by the BCA method (CAS No. P0010S, Beyotime, Shanghai, China), and protein samples were denatured with 5 × protein sample buffer (CAS No. P0015L, Beyotime, Shanghai, China). Thereafter, 20 µg of denatured protein samples were separated on a FuturePAGE™ 4–20% protein separation gel (ACE Biotechnology, Nanjing, China) at 130 V for 60 min, proteins were transferred to PVDF membranes (Merck Millipore, Darmstadt, Germany), and membranes were washed three times with Tris-buffered saline with Tween-20 (TBST) for 10 min and blocked with 5% skimmed milk (Sangong, Shanghai, China) for 1 h at room temperature. After blocking, membranes were incubated with rabbit polyclonal antibodies against SOD1 (WL01846, 1:1500), SOD2 (WL02506, 1:1500), CHOP (WL00880, 1:1500), and Bcl2 (WL01556, 1:500) (Wanleibio, Shenyang, China); GCLM (ET1705-87, Hua’an Biological, Hangzhou, China); GRP78 (11587-1-AP, 1:5000), ATF4 (10835-1-AP, 1:800), PERK (20582-1-AP, 1:1000), VDAC1 (10866-1-AP, 1:2000), MFN2 (12186-1-AP, 1:10,000), BAX ( 50599-2-Ig, 1:3000), Cleaved-caspase3 (25128-1-AP, 1:1000), and β-actin (66009-1-Ig, 1:30,000) (Proteintech, Wuhan, China); P-PERK (bs-3330R, 1:1000, Bioss, Beijing, China); MCU (A21753, 1:1000, ABclonal, Wuhan, China); and β-tubulin (AP0064, 1:5000, Annoron, Beijing, China) overnight at 4 °C and with secondary antibodies for 2 h at room temperature. Signals were detected using an enhanced chemiluminescence detection kit (Vazyme, Nanjing, China) and visualized using a chemiluminescence imaging system (Shanghai Qinxiang Scientific Instrument, Shanghai, China). A densitometric analysis of protein bands was performed using ImageJ 1.8.0 software.

### 2.6. Measurement of Indicators of OS

After treatment, GCs were collected, washed twice with PBS, resuspended in 400 µL of PBS, and pulverized using an ultrasonic cell grinder (Scientz, Ningbo, China) in an ice bath. The pulverization was performed with 5 cycles of 3 s each time with an interval of 30 s, at a power of 300 W. The supernatant was collected after centrifuged at 4 °C and 12,000× *g* for 10 min. Catalase (CAT, A007-1-1) activity, total superoxide dismutase (T-SOD, A001-1) activity, and the malondialdehyde (MDA, A003-1) content were determined using kits provided by Nanjing Jiancheng Bioengineering Institute (Nanjing, China).

### 2.7. Transmission Electron Microscopy

After treatment, GCs were collected and immediately fixed with 2.5% glutaraldehyde solution at 4 °C for 24 h. Subsequently, cells were post-fixed with 1% osmium tetroxide, subjected to gradient ethanol dehydration, and embedded in epoxy resin at a ratio of 1:1 with propylene oxide. Sections were imaged using a JEM1400 transmission electron microscope (JEOL, Tokyo, Japan) to observe mitochondrial and ER morphology.

### 2.8. ATP Content Analysis

The ATP content of GCs was quantified using an ATP assay kit (CAS No. S0026, Beyotime, Shanghai, China). After treatment, cells were lysed with 100 µL of lysis solution and centrifuged at 1200× *g* at 4 °C for 5 min. The supernatant was collected and 50 µL of ATP assay working solution was added to determine the ATP content. RLU values were measured using a GloMax 20/20 luminescence detector (Promega, Madison, WI, USA). The protein concentration was determined by the BCA method and the ATP content was calculated.

### 2.9. Measurement of the NAD^+^/NADH Ratio

The NAD^+^ content and NAD^+^/NADH ratio in GCs were determined using an NAD^+^/NADH assay kit (WST-8 method) (CAS No. S0175, Beyotime, Shanghai, China). After treatment, cells were lysed with 200 µL of NAD^+^/NADH extract and collected. Lysed cells were centrifuged at 1200× *g* for 5 min at 4 °C. The supernatant was collected and the NAD^+^ content and NAD^+^/NADH ratio were determined using the instruction manual. The NAD^+^ content was determined using the following formula: [NAD^+^] = [NADtotal] − [NADH]. The NAD^+^/NADH ratio was determined using the following formula: [NAD^+^]/[NADH] = ([NADtotal] − [NADH])/[NADH].

### 2.10. Determination of the MMP

The MMP of GCs was assessed using the JC-1 fluorescent probe (CAS No. C2003S, Beyotime, Shanghai, China). Briefly, after cell treatment, cells were washed twice with PBS. Then, the cells were incubated with 1 mL of JC-1 staining working solution (5 μg/mL in serum-free medium) for 20 min at 37 °C. Thereafter, the supernatant was removed, and cells were washed twice with JC-1 staining working solution. Images were captured using an IX73 inverted microscope (Olympus, Tokyo, Japan). Quantitative analysis was performed using ImageJ 1.8.0 software.

### 2.11. Analysis of Mitochondrial ROS

MitoSOX Red (CAS No. Hyd1055, MCE, Shanghai, China) working solution was prepared using pre-warmed serum-free medium. Cells were incubated with 500 µL/well of this solution for 20 min at room temperature, washed with medium three times for 5 min each time, and observed under an inverted fluorescence microscope (IX73, Olympus, Tokyo, Japan). Quantitative analysis was performed using ImageJ 1.8.0 software.

### 2.12. Measurement of Ca^2+^ Content

Ca^2+^ levels in GCs were measured using the Rhod-2/AM fluorescent probe (CAS No. 40776ES50, Yeasen, Shanghai, China) according to the manufacturer’s instructions. After treatment, cells were incubated with 4 µM Rhod-2/AM for 30 min at 37 °C while protected from light, washed three times with HBSS buffer (free of calcium and magnesium ions) (Yeasen, Shanghai, China), and observed under an inverted fluorescence microscope (IX73, Olympus, Tokyo, Japan). Quantitative analysis was performed using ImageJ 1.8.0 software.

### 2.13. Immunofluorescence Staining

GCs were fixed with 4% paraformaldehyde for 30 min, permeabilized with 1% Triton X-100 solution for 30 min, blocked with 1% bovine serum albumin for 1 h, and then incubated with a rabbit polyclonal anti-ATF4 antibody (1:150) at 4 °C overnight. Thereafter, GCs were washed with PBS, incubated with a secondary antibody (1:1000) at room temperature while protected from light for 1 h, and stained with DAPI (Beyotime, Shanghai, China) for 5 min. Finally, samples were observed using a fluorescence microscope (IX73, Olympus, Tokyo, Japan) and images were acquired.

### 2.14. Small Interfering RNA (siRNA)

Porcine ATF4-targeting siRNA (siATF4) and non-targeting negative control (NC) siRNA (Sangon, Shanghai, China) were used. The corresponding sequences are shown in Table 2. Briefly, GCs were treated with 1-DNJ for 24 h and then transfected with siATF4 or NC siRNA for 24 h using LipoRNAi™ transfection reagent (Beyotime, Shanghai, China) according to the instruction manual. Finally, GCs were treated with H_2_O_2_ for 6 h and collected for subsequent analysis.

### 2.15. Statistical Analysis

All data were collected from at least three independent experiments. Data were expressed as mean ± standard error. Experimental data were analyzed using GraphPad Prism 9.0 (GraphPad Inc., San Diego, CA, USA) and SPSS 27 software package (SPSS Inc., Chicago, IL, USA). One-way analysis of variance (ANOVA) was used for comparing three or more groups (followed by Tukey’s post hoc multiple comparison test). Student’s *t*-test was used to compare the two sets of data.

## 3. Results

### 3.1. Modeling of OS in GCs In Vitro

**Figure 1:**
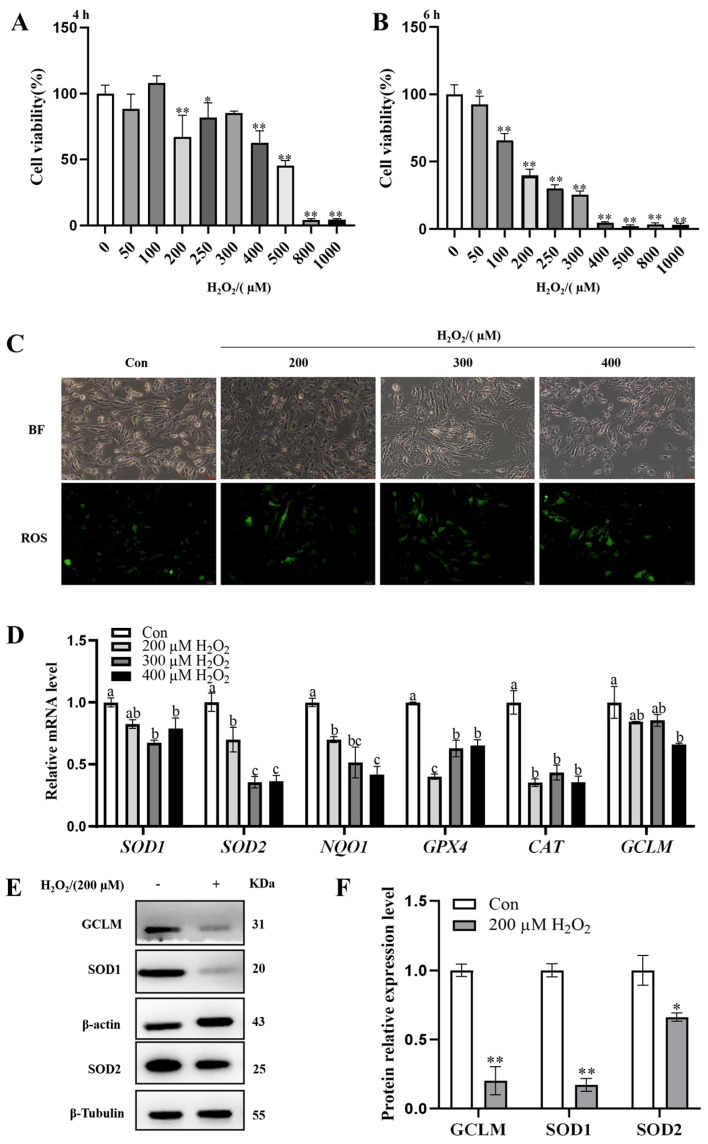
Modeling of OS in GCs in vitro. (**A**,**B**) Cellular activity of GCs after treatment with different concentrations of H_2_O_2_ (0, 50, 100, 200, 250, 300, 400, 500, 800, and 1000 µM) for 4 or 6 h, respectively, was determined using the CCK8 kit; (**C**) a DCFH-DA fluorescent probe was used to detect the ROS accumulation in GCs 6 h after treatment with 0, 200, 300, and 400 µM of H_2_O_2_. The scale bar of the graph is 50 µm and the magnification is 400×. (**D**) qRT-PCR was used to detect mRNA expression of OS-related genes in GCs induced by 0, 200, 300, and 400 µM of H_2_O_2_ for 6 h; (**E**,**F**) Western blot was used to detect the protein expression levels of GCLM, SOD1, and SOD2 in GCs induced by 0 and 200 µM H_2_O_2_ for 6 h. ^a–c^ There are no significant differences between groups with the same superscript letter, while there are significant differences between groups with different lowercase superscript letters (*p* < 0.05, ANOVA). * *p* < 0.05 and ** *p* < 0.01 compared with the Con group (Student’s *t*-test).

GCs were treated with different concentrations of H_2_O_2_ for 4 or 6 h. The CCK8 assay showed that treatment with 200 µM H_2_O_2_ for 6 h reduced GC viability to 50% compared with the control (Figure 1A,B). Treatment with H_2_O_2_ (≥200 µM) increased ROS production compared with the control (Figure 1C). Treatment with 200 µM H_2_O_2_ significantly down-regulated the mRNA expression of *SOD2*, *NQO1*, *GPX4*, and *CAT*, and down-regulated the mRNA expression of *SOD1* and *GCLM*, though the difference was not significant. Treatment with 300 and 400 µM H_2_O_2_ significantly down-regulated the mRNA expression of *SOD1*, *SOD2*, *NQO1*, *GPX4*, and *CAT* (Figure 1D). Meanwhile, treatment with 200 µM H_2_O_2_ significantly down-regulated the protein expression of SOD1, SOD2, and GCLM (Figure 1E,F). In summary, the optimal concentration and duration of H_2_O_2_ treatment to induce OS in GCs were 200 µM and 6 h.

### 3.2. Effect of 1-DNJ on the Viability and Antioxidant Activity of GCs

**Figure 2:**
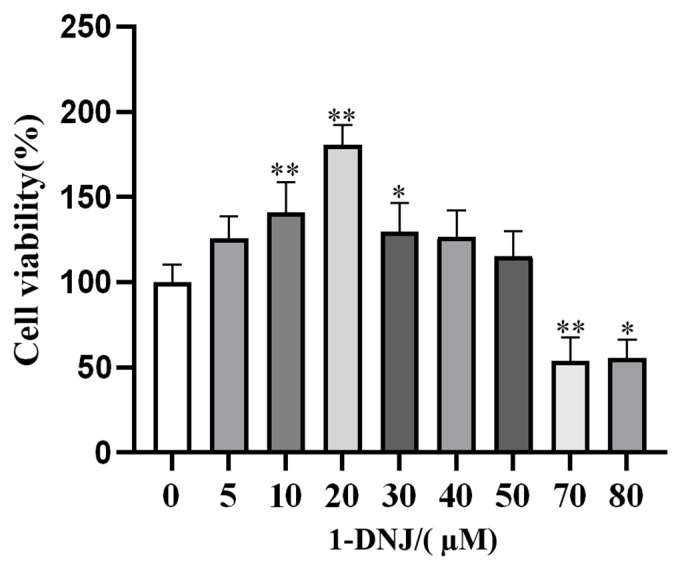
Effect of 1-DNJ on cell viability of GCs. Cellular viability of GCs treated with different concentrations of 1-DNJ (0, 5, 10, 20, 30, 40, 50, 70, and 80 µM) for 24 h was determined using the CCK8 kit. * *p* < 0.05 and ** *p* < 0.01 compared with 0 µM of 1-DNJ (Student’s *t*-test).

**Figure 3:**
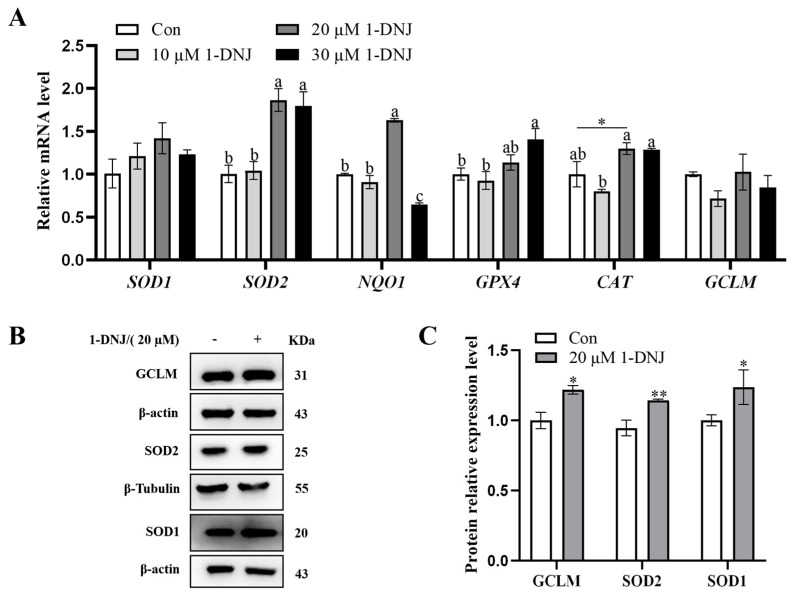
1-DNJ antioxidant analysis in GCs. (**A**) qRT-PCR was used to detect the mRNA expression of OS-related genes in GCs treated with 0, 10, 20, and 30 µM of 1-DNJ for 24 h; (**B**,**C**) Western blot detection of SOD1, SOD2, and GCLM protein expression levels of GCs at 0 and 20 µM of 1-DNJ treatment for 24 h. ^a–c^ There are no significant differences between groups with the same superscript letter, while there are significant differences between groups with different lowercase superscript letters (*p* < 0.05, ANOVA). Compared with the Con group, * *p* < 0.05 and ** *p* < 0.01 (Student’s *t*-test).

The pretreatment of GCs with various concentrations of 1-DNJ for 24 h revealed that 10–30 µM of 1-DNJ significantly increased the viability of GCs (Figure 2). qRT-PCR showed that the expression of antioxidant-related genes did not significantly differ between the 10 µM 1-DNJ-treated and control groups. Treatment with 20 µM 1-DNJ significantly increased the mRNA expression of *SOD2*, *NQO1*, and *CAT*, and increased the mRNA expression of *SOD1* and *GPX4*, though the difference was not significant. Treatment with 30 µM of 1-DNJ significantly elevated the mRNA expression of *SOD2* and *GPX4*, and increased the mRNA expression of *SOD1* and *CAT*, though the difference was not significant, and decreased the mRNA expression of *NQO1* and *GCLM* (Figure 3A). WB showed that treatment with 20 µM 1-DNJ significantly up-regulated the protein expression of SOD1, SOD2, and GCLM compared with the control group (Figure 3B,C). In summary, the optimal antioxidant effect of 1-DNJ on GCs was observed at a concentration of 20 µM after a 24 h treatment.

### 3.3. Effect of 1-DNJ on H_2_O_2_-Induced OS in GCs

**Figure 4:**
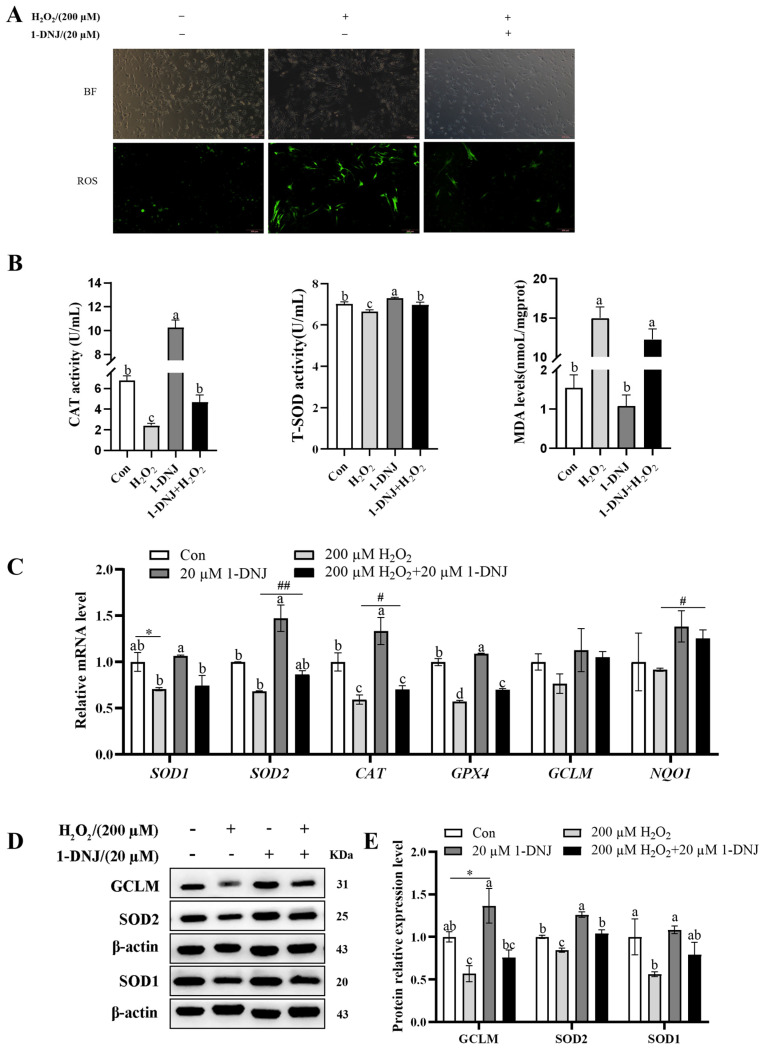
1-DNJ inhibits ROS release and enhances antioxidant defense of GCs. GCs were first treated with 1-DNJ (20 µM) for 24 h and then with H_2_O_2_ (200 µM) for 6 h. (**A**) ROS accumulation in GCs was detected by using a DCFH-DA fluorescent probe. The scale bar was 200 µm and the magnification was 100×. (**B**) CAT and T-SOD activities and MDA content of GCs were determined using kits, respectively. (**C**) qRT-PCR was used to detect mRNA expression of OS-related genes in GCs. (**D**,**E**) Western blot was performed to detect the protein expression levels of SOD1, SOD2, and GCLM in GCs. ^a–d^ There are no significant differences between groups with the same superscript letter, while there are significant differences between groups with different lowercase superscript letters (*p* < 0.05, ANOVA). Compared with the Con group, * *p* < 0.05 (Student’s *t*-test); compared with the H_2_O_2_ group, ^#^ *p* < 0.05 and ^##^ *p* < 0.01 (Student’s *t*-test).

**Figure 5:**
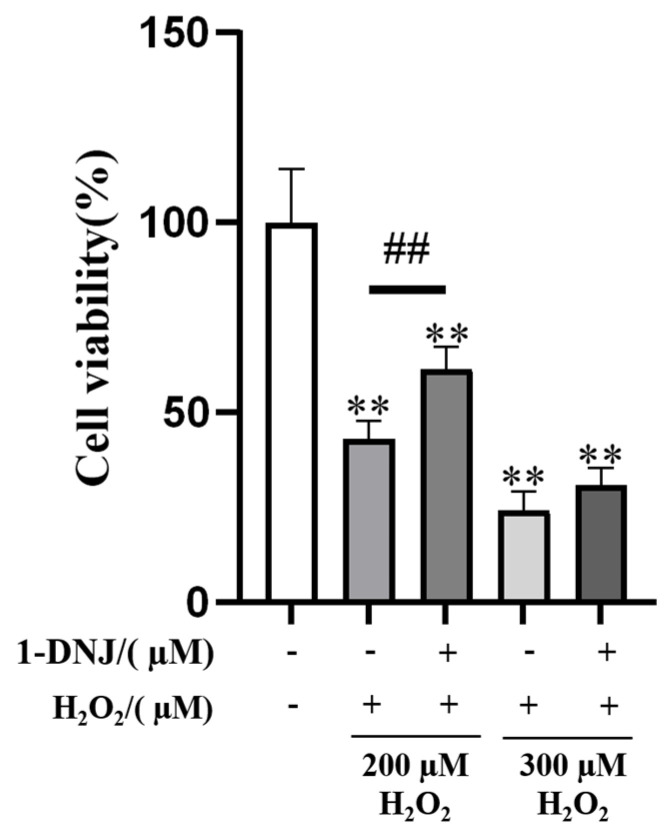
Effect of 1-DNJ on cell viability reduced by H_2_O_2_. The GCs were first pretreated with 20 µM 1-DNJ for 24 h, and then induced with 200 and 300 µM, respectively, of H_2_O_2_ for 6 h. The cell activity was determined using the CCK8 kit. Compared with the Con group, ** *p* < 0.01 (Student’s *t*-test); compared with the H_2_O_2_ group,^##^ *p* < 0.01 (Student’s *t*-test).

The ROS content was obviously lower in the co-processing group than in the H_2_O_2_-treated group (Figure 4A). Meanwhile, the co-processing group significantly elevated the H_2_O_2_-induced down-regulation of T-SOD and CAT activities and decreased the H_2_O_2_-induced elevation of MDA content, though the difference was not significant (Figure 4B). Compared with the H_2_O_2_ group, the expression of *SOD2*, *GPX4*, *CAT*, and *NQO1* mRNA was significantly higher in the co-processing group, and the expression of *SOD1* and *GCLM* mRNA was elevated, but the difference was not significant (Figure 4C). WB showed that 1-DNJ significantly elevated the H_2_O_2_-induced down-regulation of SOD2 protein expression and elevated the H_2_O_2_-induced down-regulation of SOD1 and GCLM protein expression, though the difference was not significant (Figure 4D,E). Co-treatment with 20 µM 1-DNJ and 200 µM H_2_O_2_ significantly augmented the H_2_O_2_-induced reduction of GCs viability (Figure 5). In summary, 1-DNJ mitigated H_2_O_2_-induced oxidative damage in GCs.

### 3.4. Effect of 1-DNJ on the Ultrastructures of Mitochondria and ER in H_2_O_2_-Treated GCs

**Figure 6:**
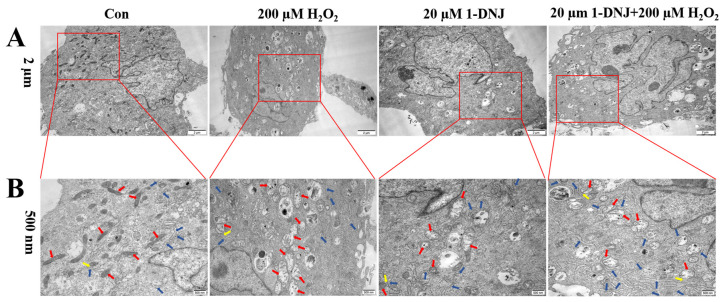
Structures of mitochondria and ER ultrastructure within GCs collected under transmission electron microscopy. GCs were first treated with 1-DNJ (20 µM) for 24 h and then with H_2_O_2_ (200 µM) for 6 h. Red arrows indicate mitochondria; blue arrows indicate ER, yellow arrows indicate MAMs. Scale bars: (**A**) is 2 µm and (**B**) is 500 nm.

Transmission electron microscopy showed that the ultrastructures of mitochondria and the ER markedly differed between the H_2_O_2_-treated and control groups (Figure 6). Compared with the con group, mitochondria in the H_2_O_2_-treated group were swollen and cristae disappeared, the ER was swollen, with a short tubular shape and reduced ribosome attachment. Additionally, the distance between the ER and mitochondria was altered. In contrast, the morphologies of mitochondria and the ER, as well as the distance between these organelles, were normalized in the co-processing group. Therefore, this study tentatively concludes that H_2_O_2_ primarily exerts its cytotoxic effects through the induction of OS-mediated damage to the ER and mitochondria.

### 3.5. Effect of 1-DNJ on OS-Induced Mitochondrial Dysfunction in GCs

**Figure 7:**
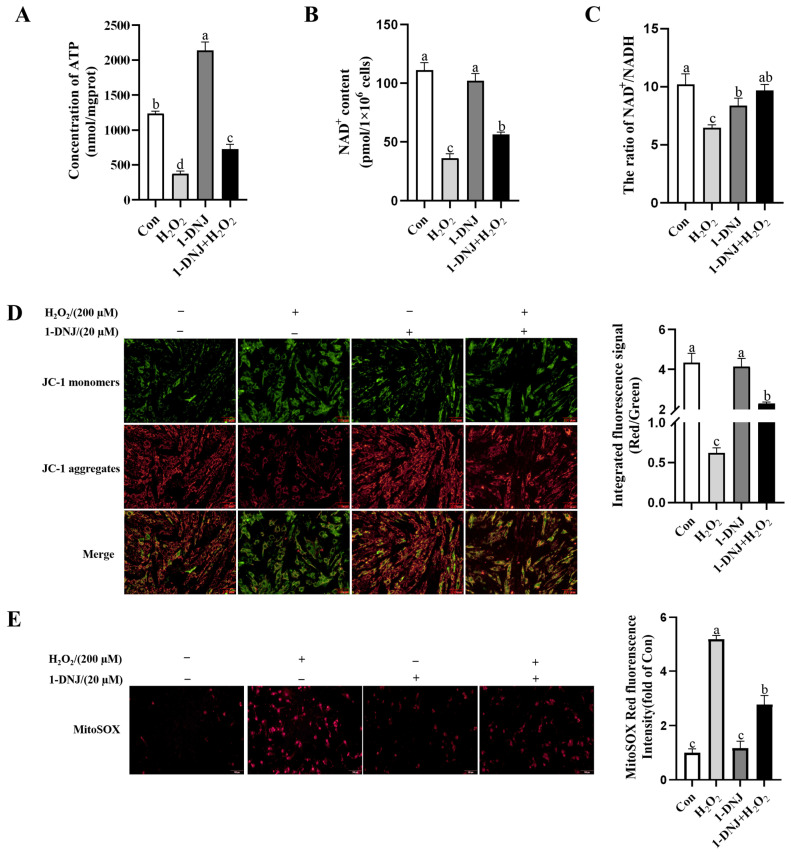
Effect of 1-DNJ on H_2_O_2_-induced mitochondrial dysfunction in GCs. GCs were first treated with 1-DNJ (20 µM) for 24 h and then with H_2_O_2_ (200 µM) for 6 h. (**A**) ATP content in GCs was detected using an ATP assay kit. (**B**,**C**) The content of NAD^+^ in GCs and the NAD^+^/NADH ratio was detected using an NAD^+^/NADH assay kit. (**D**) Changes in MMP in GCs were assessed with the fluorescent probe JC-1. (**E**) Detection of mtROS levels within GCs was assessed using the MitoSOX Red fluorescent probe. (**D**,**E**) were scaled to 100 µm and magnified to 200×. ^a–d^ There are no significant differences between groups with the same superscript letter, while there are significant differences between groups with different lowercase superscript letters (*p* < 0.05, ANOVA).

Mitochondria are important organelles for metabolism in GCs and have an important influence on the regulation of ATP synthesis, apoptosis, and antioxidant effects. The ATP and NAD^+^ contents and the NAD^+^/NADH ratio were significantly lower in the H_2_O_2_-treated group than in the con group, while 1-DNJ significantly increased these H_2_O_2_-induced reductions (Figure 7A–C). When assessing changes of the MMP, red fluorescence representing the JC-1 polymer was significantly lower in the H_2_O_2_-treated group than in the con group; however, red fluorescence was increased and green fluorescence was reduced in the remission group (Figure 7D), indicating that 1-DNJ alleviates the H_2_O_2_-induced reduction of the MMP. In addition, the red fluorescence indicative of mitochondrial ROS was higher in the H_2_O_2_-treated group compared to the con group, indicating the presence of OS and its detrimental effects on mitochondria (Figure 7E); however, 1-DNJ significantly attenuated this effect. In conclusion, 1-DNJ improves H_2_O_2_-induced mitochondrial dysfunction.

### 3.6. Effect of 1-DNJ on OS-Induced Apoptosis of GCs

**Figure 8:**
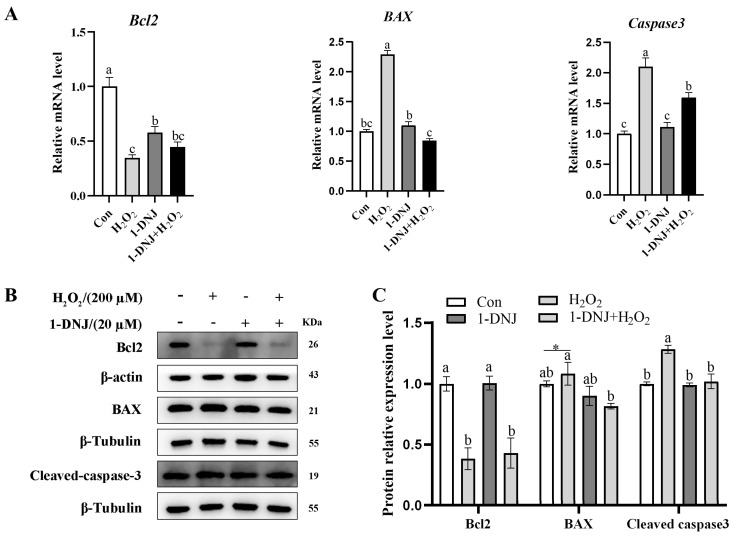
Effect of 1-DNJ on OS-induced apoptosis of GCs. GCs were first treated with 1-DNJ (20 µM) for 24 h and then with H_2_O_2_ (200 µM) for 6 h. (**A**) qRT-PCR was used to detect the mRNA expression of apoptosis-related genes. (**B**,**C**) Western blot was used to detect the expression levels of apoptotic proteins: BAX, Bcl2, and Cleaved-caspase3. ^a–c^ There are no significant differences between groups with the same superscript letter, while there are significant differences between groups with different lowercase superscript letters (*p* < 0.05, ANOVA). Compared with the Con group, * *p* < 0.05 (Student’s *t*-test).

The apoptosis of GCs was detected by WB and qRT-PCR; the results showed that the mRNA and protein expression of Bcl2 was significantly reduced in the H_2_O_2_-treated group. In contrast, the mRNA expression of *BAX* and *Caspase3* significantly increased, and the protein expression of BAX and Cleaved-caspase3 significantly increased; however, 1-DNJ attenuated these effects (Figure 8A–C). In summary, H_2_O_2_-induced OS can trigger mitochondrial dysfunction, leading to the development of apoptosis, and 1-DNJ is able to alleviate this series of changes.

### 3.7. Effect of 1-DNJ on MAMs in OS-Treated GCs

**Figure 9:**
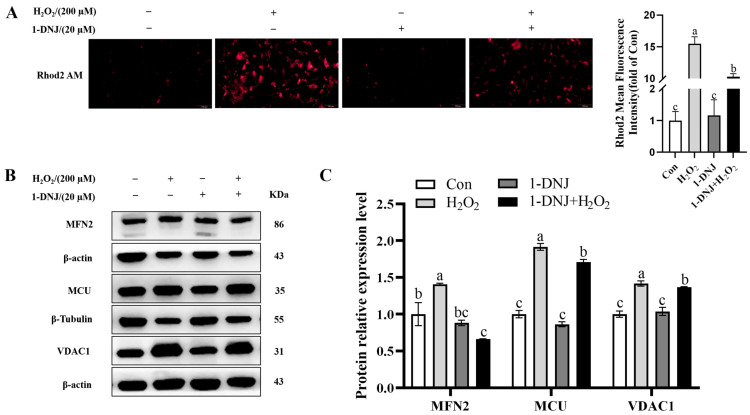
Effect of 1-DNJ on the function of MAMs in GCs by OS. GCs were first treated with 1-DNJ (20 µM) for 24 h and then with H_2_O_2_ (200 µM) for 6 h. (**A**) Ca^2+^ content in GCs was measured using the fluorescent probe Rhod-2/AM, the scale bar was 100 µm and the magnification was 200 ×. (**B**,**C**) Western blot was used to detect the expression levels of MAMs-representative proteins MCU, MFN2, and VDAC1. ^a–c^ There are no significant differences between groups with the same superscript letter, while there are significant differences between groups with different lowercase superscript letters (*p* < 0.05, ANOVA).

The ER and mitochondria are interconnected organelles with contact sites called MAMs. Recent studies showed that MAMs provide new avenues to explaining the mutual regulation of ERS and mitochondrial dysfunction. The mitochondrial Ca^2+^ content was significantly higher in the H_2_O_2_-treated group than in the con group, whereas 1-DNJ significantly abrogated this effect (Figure 9A). The protein expression of MCU, MFN2, and VDAC1 were significantly higher in the H_2_O_2_-treated group than in the con group (Figure 9B,C), whereas 1-DNJ could significantly mitigate these changes induced by H_2_O_2_. In summary, 1-DNJ affected the integrity of MAMs in response to OS damage.

### 3.8. Effect of 1-DNJ on OS-Induced ERS in GCs

**Figure 10:**
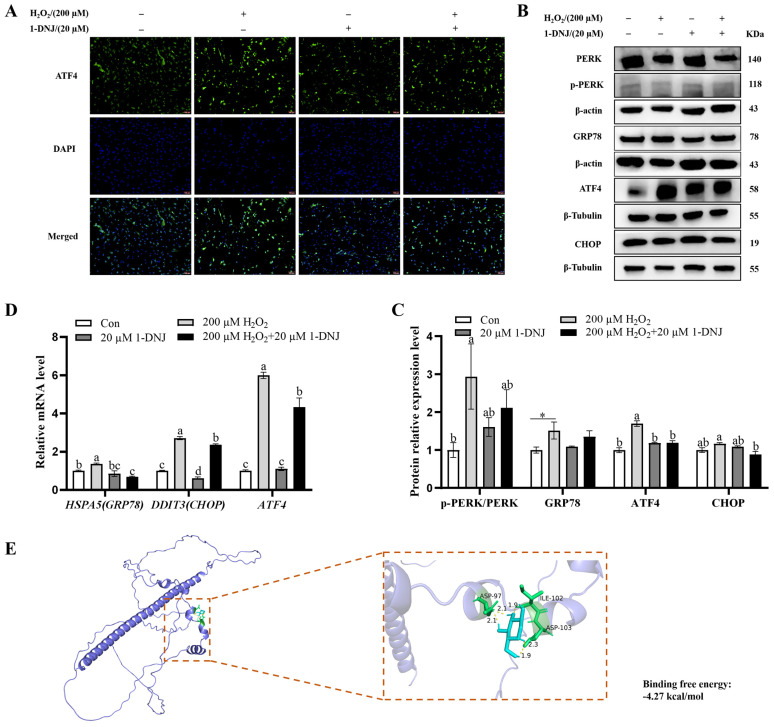
Effect of 1-DNJ on OS-induced endoplasmic reticulum stress response in GCs. GCs were first treated with 1-DNJ (20 µM) for 24 h and then with H_2_O_2_ (200 µM) for 6 h. (**A**) Changes in ATF4 expression in GCs were detected by immunofluorescence; (**B**,**C**) Western blot detection of p-PERK/PERK, GRP78, ATF4, and CHOP protein expression; and (**D**) qRT-PCR detection of *GRP78*, *ATF4*, and *CHOP* mRNA expression. (**E**) Molecular docking simulation for the ligand−protein binding of 1-DNJ with ATF4. (**A**) Scale bar is 200 µm and magnification is 100×. ^a–d^ There are no significant differences between groups with the same superscript letter, while there are significant differences between groups with different lowercase superscript letters (*p* < 0.05, ANOVA). Compared with the Con group, * *p* < 0.05 (Student’s *t*-test).

To determine whether H_2_O_2_-induced OS is related to ERS, the effect of 1-DNJ on ERS responses, particularly on the PERK-ATF4 pathway of the UPR, was determined. Immunofluorescence analysis revealed that ATF4 expression was higher in the H_2_O_2_-treated group than in the control group, whereas 1-DNJ attenuated the H_2_O_2_-induced elevation of ATF4 expression (Figure 10A). In addition, compared with the control group, H_2_O_2_ significantly elevated the mRNA expression of ATF4, CHOP, and GRP78 and significantly increased the protein expression of p-PERK/PERK, GRP78, and ATF4. Conversely, 1-DNJ significantly attenuated the effects of H_2_O_2_ on the PERK-ATF4 pathway (Figure 10B–D). To confirm the possible interaction between 1-DNJ and ATF4 at the molecular level, we carried out the protein–ligand docking analysis, which suggested a stable combination (Figure 10E). The results indicated that H_2_O_2_-induced OS significantly provoked ERS, a process primarily mediated by the activation of the PERK-ATF4 signaling pathway within the endoplasmic reticulum’s UPR.

### 3.9. Crosstalk Between ERS, OS, and Mitochondrial Dysfunction

**Figure 11:**
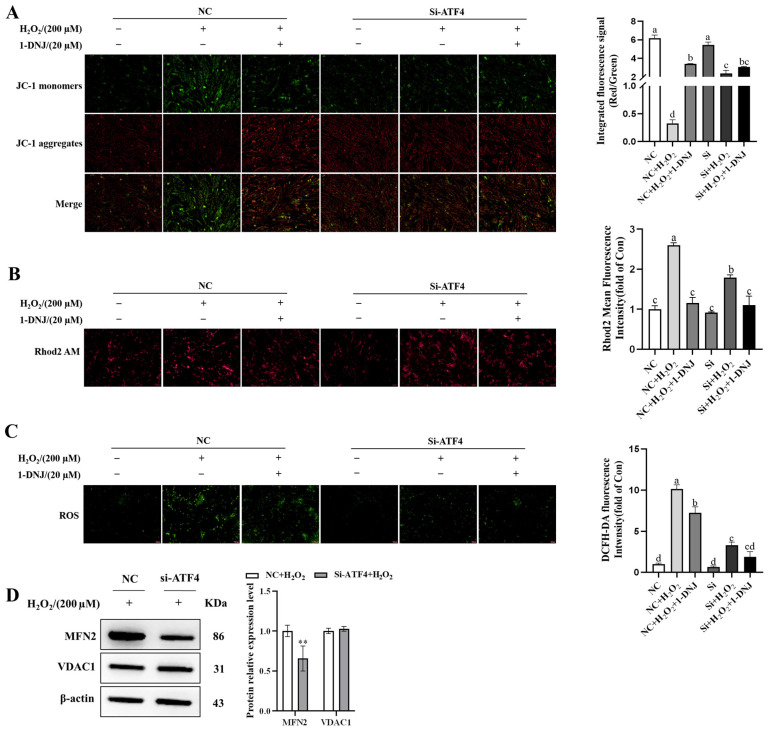
Crosstalk between ERS, OS, and mitochondrial dysfunction. GCs were first treated with 1-DNJ (20 µM) for 24 h, followed by the transfection of ATF4 knockdown for 24 h, and then exposed to H_2_O_2_ (200 µM) for 6 h. (**A**) MMP changes within GCs were detected using JC-1, the fluorescent probe. (**B**) Ca^2+^ content in GCs was measured using the Rhod-2/AM fluorescent probe. (**C**) ROS accumulation in GCs was detected using the DCFH-DA fluorescent probe. (**D**) WB analysis was used to detect the expression levels of MAMs-representative proteins. (**A**–**C**) The scale bar is 100 µm and the magnification is 200×. ^a–d^ There are no significant differences between groups with the same superscript letter, while there are significant differences between groups with different lowercase superscript letters (*p* < 0.05, ANOVA). Compared with the NC + H_2_O_2_ group, ** *p* < 0.01 (Student’s *t*-test).

OS is associated with cellular homeostasis and apoptosis. Given that ROS can promote protein misfolding and lead to ERS, we hypothesized that H_2_O_2_-induced mitochondrial dysfunction is responsible for OS-mediated ERS. Therefore, we explored whether ATF4 can mediate alterations in MAMs. The knockdown efficiency of ATF4 was first verified. ATF4-812 demonstrated better knockdown efficiency than the other siRNAs based on the determination of mRNA expression levels after transfection (Supplementary Figure S1). The knockdown of ATF4 significantly alleviated the OS-induced decrease in MMP (Figure 11A). Intracellular Ca^2+^ content was significantly attenuated in ATF4-knockdown cells compared to the H_2_O_2_ treatment group (Figure 11B). The level of intracellular ROS was much lower in ATF4-knockdown cells than those in the H_2_O_2_ treatment group (Figure 11C). WB analysis showed that ATF4-knockdown significantly repressed the expression levels of MAMs-representative proteins MFN2 compared with the H_2_O_2_ group (Figure 11D). In summary, ATF4 plays an important role in regulating the interaction between ERS, OS, and mitochondrial dysfunction. The knockdown of ATF4 attenuates cellular damage from OS by affecting mitochondrial function.

## 4. Discussion

**Figure 12:**
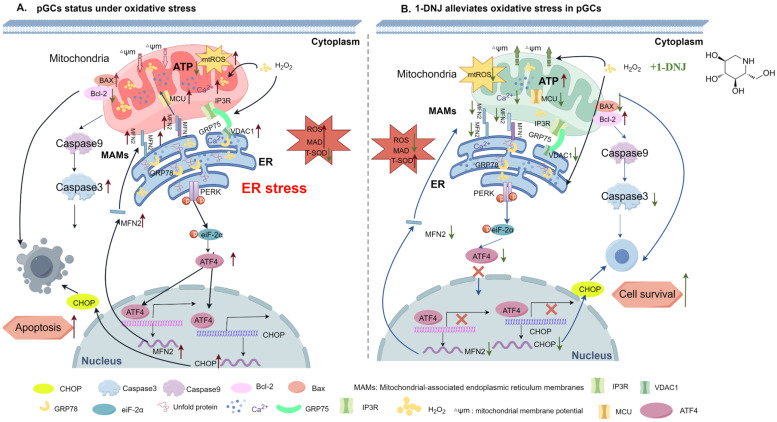
Mechanism of 1-DNJ alleviating OS in porcine GCs. (**A**) When OS occurs in porcine GCs, the ERS pathway is activated, leading to ER damage and the triggering of ERS. Meanwhile, mitochondrial function is impaired and the level of mitochondrial OS is increased. During this process, OS affects the communication between the mitochondria and ER through MAMs, resulting in an increase in the content of Ca^2+^ in the mitochondria and changes in the activities of apoptosis-related proteins Bax, Bcl2, and Caspase3. Ultimately, this leads to an increase in apoptosis of porcine GCs. (**B**) When OS occurs in GCs, the addition of 1-DNJ can inhibit the activity of the PERK-ATF4 signaling pathway, relieve ERS, restore ER and mitochondrial functions, prevent ER calcium inflow from the ER into the mitochondria by regulating the expression of MAMs-related proteins and MCU, reduce mitochondrial OS levels, and restore MMP and ATP synthesis. Moreover, MFN2 is a potential target gene downstream of ATF4. Ultimately, 1-DNJ promotes the expression of the anti-apoptosis-related protein Bcl2, inhibits the expression of pro-apoptosis proteins BAX and Caspase3, and finally, alleviates the apoptosis of porcine GCs caused by OS. This figure was drawn by Figdraw.

The reduced clearance or excessive production of ROS induced by environmental or metabolic factors triggers oxidative damage and apoptosis in GCs, which in turn leads to follicular atresia and associated ovulatory disorders. However, the molecular mechanisms underlying OS-induced ROS production and apoptosis in GCs have not been fully revealed. To understand this issue, the present study was conducted to construct an OS model using H_2_O_2_ in GCs and to detect H_2_O_2_-mediated ERS, mitochondrial OS, and apoptosis through MAMs (Figure 12A). H_2_O_2_ induced oxidative damage, mitochondrial dysfunction, ERS, and the disruption of the integrity and function of MAMs in GCs, resulting in the apoptosis of GCs (Figure 12A), while 1-DNJ alleviated these effects. The integrity of MAMs might be disrupted via the direct interaction between the MCU-VDAC1 complex and PERK-mediated ERS (Figure 12B). These results suggest that 1-DNJ affects mitochondrial dysfunction in GCs by regulating ERS-mitochondrial OS through MAMs, thereby influencing apoptosis and follicular atresia.

OS is characterized by an imbalance between ROS production and scavenging by the antioxidant defense system, and follicular development is inevitably affected by ROS-induced OS. Zhang et al. [26] reported that the ROS-induced apoptosis of GCs is an important cause of follicular atresia. H_2_O_2_, a type of ROS, is often used to induce oxidative damage in cells in vitro because H_2_O_2_-treated cells can produce a large amount of ROS within a short period, which in turn induces cellular OS [27]. In this study, an oxidative damage model of GCs was constructed using H_2_O_2_, and cell viability was around 50% while ROS generation increased upon treatment with 200 µmol/L H_2_O_2_ for 6 h. However, when the H_2_O_2_ concentration exceeded 200 µmol/L, the apoptosis rate significantly increased, which was not conducive to modeling the normal apoptosis of GCs in vivo. Therefore, we constructed the oxidative damage model of GCs by performing treatment with 200 µmol/L H_2_O_2_ for 6 h. This condition is consistent with the modeling condition of Li et al. [28], but Kong et al. [29] chose the optimal concentration of 100 µmol/L and the optimal duration of 6 h for a model of OS induced by H_2_O_2_. The reason for this difference might be the different feeding conditions and living environments of the pigs from which ovaries were collected. High altitude, poor nutrition, and intensive production can increase OS in the body [30]. Therefore, we hypothesize there are differences in feeding methods, climate, and altitude between the test pigs used in this study (Nanjing, China) and those used in Kong’s study (Dali, China) and that test pigs in Dali are located at a higher altitude and more likely to experience OS.

Antioxidant supplementation may be an effective approach to control ROS production [31] and is still being explored as a potential strategy to overcome reproductive disorders associated with follicular atresia. A growing number of studies have shown that supplementation with natural antioxidants effectively inhibits oxidative damage in ovaries [26]. 1-DNJ, a major constituent of Morus alba leaf extract [32], is a potent free radical scavenger and antioxidant, with an additional role in glucose metabolism. Low levels of DNJ can improve the antioxidant level, which is beneficial to the intestinal health of chickens [20]. Li et al. [22] found that 1-DNJ alleviates lipopolysaccharide-induced ROS accumulation in rat cardiomyocytes. In the present study, GCs treated with 200 µmol/L of H_2_O_2_ displayed a significantly decreased mRNA expression of *SOD1*, *GPX4*, and *CAT*, and decreased mRNA expression of *SOD2*, *GCLM*, and *NQO1*, but the difference was not significant; they also displayed a significantly decreased protein expression of SOD1, SOD2, and GCLM. The level of OS was decreased after 1-DNJ treatment, especially at a concentration of 20 µmol/L, which protected against oxidative damage by enhancing the antioxidant capacity of H_2_O_2_-treated GCs. 1-DNJ reduces the cellular damage caused by free radicals by reducing their production, thereby improving cell viability. These results suggest that H_2_O_2_ induces OS in GCs by disrupting the antioxidant enzyme system, while 1-DNJ alleviates oxidative damage.

Mitochondria are one of the main sites of ROS production. Excessive ROS accumulation can damage mitochondria and affect their function. As the key to energy metabolism, mitochondria provide 90% of ATP to the cell [33]. ATP, as an important intracellular energy carrier, plays an important role in a variety of physiological processes, such as hormone synthesis, follicular development, and ovulation. These processes require a large amount of ATP. A decrease in ATP levels indicates that mitochondria, the “powerhouse of the cell”, have been damaged. At the same time, the NAD^+^/NADH ratio directly regulates ATP production. When the energy demand increases, NADH oxidizes to generate NAD^+^, supporting ATP synthesis. Conversely, when the energy demand decreases, the accumulation of NADH inhibits metabolic pathways, such as glycolysis, and reduces ATP production [34]. The MMP reflects the functional and metabolic state of the mitochondria. Excessive ROS can induce the opening of mitochondrial osmotic transition pores, resulting in a decrease in the MMP, which is an important molecular marker of the OS state of mitochondria [35]. In HT22 cells, the removal of ROS can reverse the AgNP-induced decrease in the MMP and ATP level [36]. A decrease in the MMP is a hallmark of mitochondrial dysfunction and is closely linked to apoptosis. Stephanie et al. [23] found that the ROS accumulation is associated with elevated apoptosis and a decreased MMP in H_2_O_2_-treated SH-SY5Y cells, consistent with the present study. By focusing on mitochondrial-associated proteins, OPA1 was identified as a target for 1-DNJ. 1-DNJ stabilizes OPA1 oligomers, enhances MMP, and promotes ATP production, which is consistent with its role as a major regulator of cristae formation and the functional assembly of respiratory chain complexes. In the present study, we found that 1-DNJ alleviated the H_2_O_2_-induced reductions of the MMP and ATP level. Normal mitochondrial structure is critical for mitochondrial function. Mitochondrial fragmentation, cristae loss, and membrane rupture, as well as rupture reduce ATP synthesis and trigger cell death [37]. Transmission electron microscopy showed that H_2_O_2_ induced morphological mitochondrial aberrations, which were characterized by mitochondrial swelling and the loss of cristae. In summary, H_2_O_2_ triggers the mitochondrial apoptotic pathway by elevating ROS levels in GCs, thereby disrupting the mitochondrial morphology and mitochondrial dysfunction (via altered mitochondrial ROS, MMP, and ATP levels). However, 1-DNJ can alleviate the mitochondrial damage of GCs induced by H_2_O_2_. Although 1-DNJ cannot directly counteract the effect of H_2_O_2_, it can protect cells by restoring mitochondrial function.

Research conducted in vivo has confirmed that OS caused by the excessive generation of ROS triggers the apoptosis of GCs [37]. By reducing antioxidant defense and up-regulating apoptotic proteins, follicular ROS induce apoptosis and accelerate follicular atresia [38]. Similarly, ROS from diverse sources can induce apoptosis in follicular GCs [38,39]. In this study, H_2_O_2_ induced mitochondria damage via increased ROS levels. The protein expression of Cleaved-caspase3, the “executor” of apoptosis, along with both protein and mRNA expression of the pro-apoptotic protein BAX were significantly increased. Conversely, Bcl2, an anti-apoptotic protein, was down-regulated in H_2_O_2_-treated cells. 1-DNJ reduced mitochondrial apoptosis induced by H_2_O_2_. These results indicate that H_2_O_2_ induces OS in GCs, activating the mitochondrial apoptosis pathway, while 1-DNJ alleviates this effect.

Intracellular calcium homeostasis is mainly maintained by the ER, together with mitochondria, lysosomes, other organelles, and the plasma membrane. However, stimuli such as OS, nutritional deficiencies, and chemical injuries are predisposing factors that reduce calcium levels in the ER lumen, leading to the accumulation of unfolded or misfolded proteins in the ER lumen and inducing an ERS response [12]. ERS further exacerbates the depletion of calcium in the ER. However, excess calcium in the ER is not only released into the cytoplasm but is also transferred to mitochondria through MAMs, which affects mitochondrial function [40]. MAMs not only serve as the spatial basis for the interaction between the ER and mitochondria, but also act as a central hub for various cellular processes, such as signaling and apoptosis [17]. GRP75-VDAC1 located on MAMs facilitates the transfer of Ca^2+^ from the ER to mitochondria by inducing Ca^2+^ overload, and excess Ca^2+^ is transported across the outer mitochondrial membrane to the mitochondrial matrix via the mitochondrial calcium uniporter, further inducing apoptosis [11]. In the present study, we found that H_2_O_2_ significantly elevated the Ca^2+^ content in the mitochondria of GCs. Moreover, H_2_O_2_-induced OS in GCs significantly enhanced MCU, VDAC1, and GRP75 protein expression, whereas 1-DNJ attenuated these effects. Thus, 1-DNJ can overcome H_2_O_2_-induced OS with the disruption of the integrity and function of MAMs.

Under pathological conditions such as OS, nutritional deficiencies, and chemical injuries, the structure or function of the ER is disrupted, leading to the accumulation of unfolded or misfolded proteins in the lumen of the ER, which induces ERS [12]. ERS, a relatively newly discovered method of initiating apoptosis [41], has been used as an important tool to study the mechanisms of apoptotic cell death. Maintaining ER homeostasis through the UPR to inhibit the expression of apoptotic proteins in GCs and reduce follicular atresia is essential for follicular development and oocyte maturation. The oocyte maturation rate and embryonic development can be recovered or even improved when ERS is reduced [42]. The Bushen Jieyu Tiaochong Formula delays the ERS-induced apoptosis of GCs by inhibiting the PERK-ATF4-CHOP signaling pathway [41]. The suppression of ERS significantly increases the maturation rate and decreases the ROS level and apoptosis in bovine oocytes [43]. In the present study, we found that H_2_O_2_ elevated the protein and mRNA expression of the ERS markers GRP78, ATF4, and CHOP and protein expression of p-PERK/PERK in GCs; however, 1-DNJ attenuated the effects of H_2_O_2_ on the PERK-ATF4 pathway. In some cell types, such as IPEC-J2 cells, H_2_O_2_-induced ERS leads to a luminal enlargement of the ER, which may be an adaptive change in the cells to resist unfavorable environmental challenges such as OS [44]. In the current study, transmission electron microscopy showed that the H_2_O_2_-induced ER was swollen, with a short tubular shape, and exhibited reduced ribosome attachment. In summary, H_2_O_2_-induced OS significantly triggers ERS, and this process is primarily mediated through the activation of the PERK-ATF4 signaling pathway in response to UPR.

ATF4, a member of the cAMP-responsive element-binding protein family of basic leucine zipper proteins, is a multifunctional transcription factor involved in many cellular functions, such as amino acid transport and metabolism, the OS response, and protein homeostasis, and also induces apoptosis, cell cycle arrest, and senescence [45]. A knockdown of ATF4 in Hela cells increases the ATP content [46], suggesting that ATF4 regulates mitochondrial function. In this study, we found that siATF4 significantly attenuated the H_2_O_2_-induced elevations of MFN2 protein expression (with no effect on VDAC1 protein expression), suppressed ROS production and ER–mitochondria calcium transport, and alleviated the H_2_O_2_-induced reduction of the MMP in GCs. Therefore, ATF4 plays an important role in regulating the interaction between ERS, OS, and mitochondrial dysfunction, and its knockdown attenuates cellular damage from OS by affecting mitochondrial function and related signaling pathways.

ERS regulates mitochondrial metabolic function through MAMs. Excessive ERS disrupts mitochondrial ROS outburst and ATP synthesis, contributing to many diseases. Based on the results of this study, a 1-DNJ-targeted MAMs regulatory therapy strategy should be developed. 1-DNJ is combined with existing antioxidants (such as Nrf2 activators) or anti-inflammatory agents (such as JNK inhibitors) to synergistically enhance efficacy through multiple pathways. By restoring the mechanism of ER–mitochondrial homeostasis to relieve OS, 1-DNJ provides a novel therapeutic target for improving female mammals’ reproductive performance and reducing ovarian mitochondrial damage. The primary limitation of this study is the lack of in vivo validation, which is crucial for confirming the physiological relevance of our findings. To enhance the translational potential of these results, future research should focus on elucidating the mechanisms by which these findings can be applied in clinical settings.

## 5. Conclusions

In summary, the present study demonstrated that H_2_O_2_ induced OS in GCs, leading to ERS (the activation of the PERK-ATF4 signaling pathway), ER damage, and mitochondrial dysfunction, which further induced apoptosis. In contrast, 1-DNJ affected OS-induced damage in GCs. The mechanism by which 1-DNJ alleviates cellular oxidative damage was analyzed based on H_2_O_2_-mediated ERS and mitochondrial OS through MAMs. The results may offer a novel approach to alleviate oxidative damage in GCs, as well as provide new perspectives and potential therapeutic targets for the treatment of human ovarian diseases.

## Figures and Tables

**Table 1:** qRT-PCR primer sequences.

Gene	NCBI Number	Accession Number	Primer Sequence	Primer Length	Product Length
*GAPDH*	NM_001206359	AF017079	F: TCGGAGTGAACGGATTTGGC R: TGCCGTGGGTGGAATCATAC	20 20	147
*SOD1*	NM_001190422	AF396674	F: ATTCTGTGATCGCCCTCT R: AGCATTTCCCGTCTTTGT	18 18	119
*SOD2*	NM_214127	AF396673	F: TCTGGACAAATCTGAGCCCTAA R: TGGACGCCGACGGATACA	22 18	127
*GPX4*	NM_214407	AK232479	F: GACGACTGGCGATGTGCT R: GCTCCTGCCTCCCAAACT	18 18	232
*CAT*	XM_021081498	AK233269	F: CGAAGGCGAAGGTGTTTG R: CAAACCCACGAGGGTCAC	18 18	114
*NQO1*	NM_001159613	AK234062	F: CCTCTGGCCAATTCAGAGTGG R: CTGGATTCGGGCATCCTCTG	21 20	106
*GCLM*	XM_001926378	AK235653	F: AGAAGTGCCCGTCTACACAC R: CATCTGGAAACTCCCTGACCA	20 21	108
*Bcl2*	XM_021099593.1	AB271960	F: TCAGGGATGGGGTGAACT R: TCAGAGACAGCCAGGAGAAAT	18 21	240
*BAX*	XM_003127290	AJ606301	F: GCCGAAATGTTTGCTGAC R: GCCGATCTCGAAGGAAGT	18 18	154
*Caspase-3*	NM_214131.1	AB029345	F: TTGGACTGTGGGATTGAGACG R: CGCTGCACAAAGTGACTGGA	21 20	165
*ATF4*	XM_021090887.1	AK233046	F: TCAGACAACAGCAAGGAGGATG R: GCCAAAAGCTCATCTGGCAT	23 20	132
*GRP78*	XM_001927795.7	AK344136	F: ACCACCTACTCGTGCGTTG R: CGTCGAAGACCGTGTTCTCA	19 20	175
*CHOP*	XM_005674378.2	AK346637	F: ATTGCCTTTCTCCTTCGGGAC R: GAAGGTTTTTGACTCCTCCTCAT	21 23	139

**Table 2:** siRNA sequences used to inhibit ATF4 expression in GCs.

Target	siRNA Sequence	Sequence (5′-3′)
ATF4	SiATF4-812 SiATF4-1155 SiATF4-1251	GUCUUCCACUCCAGAUAAUTT CAGAUAAUGACAGUGGCAUTT GGAGAUUCAGUAUCUCAAATT
Control	NC	UUCUCCGAACGUGUCACGUTT

## Data Availability

The data presented in this study are available on request from the corresponding author.

## Supplementary Materials

The supporting information can be downloaded at https://www.mdpi.com/article/10.3390/antiox14040456/s1: qRT-PCR analysis showed that siATF4-812 with the strongest knockdown effects were selected for further experiments.

## Abbreviations

The following abbreviations are used in this manuscript: 1-DNJ, 1-deoxynojirimycin; ATP, adenosine triphosphate; ATF4, Activating transcription factor 4; BAX, Bcl-2-associated X protein; BCA, bicinchoninic acid; Bcl2, B-cell lymphoma 2 protein; CAT, catalase; Caspase-3, Cysteine-aspartic acid protease 3; CCK8, Cell Counting Kit-8; CHOP, C/EBP-homologous protein; GCLM, glutamate-cysteine ligase modifier subunit; GCs, granulosa cells; GPX4, Glutathione Peroxidase 4; GRP78, glucose regulated protein 78; IRE1-a, Inositol-dependent enzyme 1-a; MAMs, mitochondria-associated endoplasmic reticulum membranes; MDA, malondialdehyde; MCU, mitochondrial calcium uniporter; MFN2, mitofusin-2; MitoROS, mitochondrial reactive oxygen species; MMP, mitochondrial membrane potential; NADH, nicotinamide adenine dinucleotide; NQO1, NAD(P)H dehydrogenase, quinone 1; OPA1, Optic atrophic protein 1; OS, oxidative stress; PERK, Protein kinase R-like ER kinase; PVDF, polyvinylidene fluoride; ROS, reactive oxygen species; SDS-PAGE, sodium dodecyl sulfate-polyacrylamide gel electrophoresis; SOD, superoxide dismutase; T-SOD, total superoxide dismutase; TEM, transmission electron microscopy; TBST, Tris buffered saline with Tween 20; VDAC1, voltage-dependent anion-selective channel 1; ER, endoplasmic reticulum; ERS, endoplasmic reticulum stress; UPR, unfolded protein response.
