# Supplementary material for: Effect of 1-DNJ on Oxidative Stress-Induced Apoptosis in Porcine Ovarian GCs Through Modulation of the PERK-ATF4/MFN2 Signaling Pathway
Source: Antioxidants (Basel). 2025 Apr 11;14(4):456. doi: 10.3390/antiox14040456 (PMC12024285; doi:10.3390/antiox14040456)
Supplement: Supplementary file 1 [file antioxidants-14-00456-s001.zip › antioxidants-3535395-supplementary.pdf]

qRT-PCR analysis showed that siATF4-812 with the strongest knockdown effects were selected for further experiments.

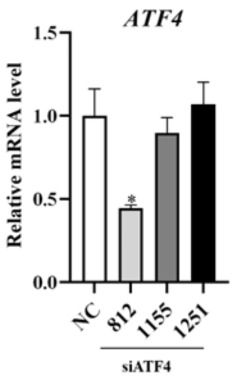

Supplementary Figure S1. Knockdown effects of ATF4. RNA interference was performed using small interfering RNAs (siRNA) directed against pig ATF4, (si-ATF4) and non-targeting negative control siRNA (NC siRNA). * $p < 0.05$ compared with the Con group.
